# Honey Environmental DNA Can Be Used to Detect and Monitor Honey Bee Pests: Development of Methods Useful to Identify *Aethina tumida* and *Galleria mellonella* Infestations

**DOI:** 10.3390/vetsci9050213

**Published:** 2022-04-27

**Authors:** Anisa Ribani, Valeria Taurisano, Valerio Joe Utzeri, Luca Fontanesi

**Affiliations:** 1 Department of Agricultural and Food Sciences, University of Bologna, Viale Giuseppe Fanin 46, 40127 Bologna, Italy; anisa.ribani2@unibo.it (A.R.); valeria.taurisano2@unibo.it (V.T.); valeriojoe.utzeri2@unibo.it (V.J.U.); 2 GRIFFA srl, Viale Fanin 48, 40127 Bologna, Italy

**Keywords:** apiculture, *Apis mellifera*, assay, beekeeping, eDNA, greater wax moth, health, mitochondrial DNA, sequencing, small hive beetle

## Abstract

Environmental DNA (eDNA) contained in honey derives from the organisms that directly and indirectly have been involved in the production process of this matrix and that have played a role in the hive ecosystems where the honey has been produced. In this study we set up PCR-based assays to detect the presence of DNA traces left in the honey by two damaging honey bee pests: the small hive beetle (*Aethina tumida*) and the greater wax moth (*Galleria mellonella*). DNA was extracted from 82 honey samples produced in Italy and amplified using two specific primer pairs that target the mitochondrial gene cytochrome oxidase I (COI) of *A. tumida* and two specific primer pairs that target the same gene in *G. mellonella*. The limit of detection was tested using sequential dilutions of the pest DNA. Only one honey sample produced in Calabria was positive for *A. tumida* whereas about 66% of all samples were positively amplified for *G. mellonella*. The use of honey eDNA could be important to establish early and effective measures to contain at the local (e.g., apiary) or regional scales these two damaging pests and, particularly for the small hive beetle, to prevent its widespread diffusion.

## 1. Introduction

Environmental DNA (eDNA) offers a way to detect organisms without direct visual observation and sampling, giving the possibility to identify and monitor cryptic and elusive organisms that would be technically difficult or time consuming to be sampled or detected [1,2,3,4]. Environmental DNA methodologies are adapted according to the organisms and the objectives of the monitoring, that, in turn, require appropriate methods of collection and analysis of specimens to produce DNA information with targeted or untargeted approaches [5,6,7]. Environmental DNA can be also useful to detect pathogens, parasites and pests that are usually very complicated to monitor, especially when there is the need to obtain timely information or when it would be important to obtain data on their dispersion [8,9].

Honey is a unique collector of eDNA that can be exploited for several applications that may benefit the beekeeping sector [10,11,12,13,14,15,16,17,18,19]. Environmental DNA in the honey derives from all organisms that directly and indirectly have been involved in its production process and that have been part of the hive ecosystem where it comes from [10,11]. For example, the pollen that is contained in the honey is a source of plant DNA that can be used to define its botanical origin and, in turn, its geographical origin [12,13]. Honey contains DNA traces of the honey bees that produced it [13,14]. This DNA has been used to identify the entomological origin of the honey [13,14,15,16,17]. Honey bee pathogens and parasites can be detected using honey eDNA, which also provides information to monitor their distribution over large geographical areas [18,19,20,21,22,23,24]. For example, we used honey eDNA to establish the first distribution map of a honey bee parasite, *Lotmaria passim*, in the north of Italy, combining geographic positioning of the production sites and a targeted analysis designed to specifically amplify DNA of this trypanosome parasite [24].

*Aethina tumida* (Murray, 1867), commonly known as small hive beetle (SHB), is a coleopter of the family Nitidulidae, that is a highly invasive and destructive scavenger of *Apis mellifera* colonies [25,26,27]. This pest is native to sub-Saharan Africa, where it is usually considered a minor problem for the local beekeeping activities due to co-evolution and adaptation of the African honey bee subspecies [26,27,28,29,30,31]. In 1996, SHB was discovered for the first time in the USA [32]. After this first identification outside its endemic range, *A. tumida* was discovered in several other countries where it is causing relevant damages to the apiculture sector [33,34,35,36,37]. The spread of SHBs is a currently ongoing and worrying process that seems difficult to face and control, as demonstrated by several recent reports that indicated the introduction of this beetle in countries of all continents, except Antarctica [36,37,38,39,40,41,42,43,44,45,46,47]. Late recognition of this pest in some newly invaded regions has prevented the possibility to establish appropriate measures to eradicate and contain SHBs, resulting in widespread infestations, impossible to manage [27,30,32,37,48]. Early detection has been suggested to be one of the most effective contingency measures to limit the spread of SHBs [37,48]. A few DNA-based detection assays applied directly to collected *A. tumida* specimens or hive debris have been proposed as monitoring tools [49,50,51,52,53,54,55].

The greater wax moth *Galleria mellonella* (Linnaeus 1758), also known as the bee-moth, or honeycomb moth, is a member of the family Pyralidae of Lepidopteran order that is considered a serious honey bee pest because of the destructive feeding habits of its larvae. Larvae of this pest usually live in beehives and feed on wax, pollen, honey and young bees, also creating a lot of damage to stored combs and beekeeping equipment [56,57]. The greater wax moth is ubiquitously distributed in all countries where beekeeping is practiced creating relevant economic losses, with increasing negative impacts especially in Africa and Asia [56,57,58,59,60]. *G. mellonella* has been mainly investigated as a model organism for insect physiology, infection mechanisms, immune response and drug testing and as a bioindicator or as a source of biotechnology applications [56,61,62,63]. As far as we know, no specifically designed DNA-based methods have been developed to detect and monitor this pest.

In this study, we designed targeted DNA-based assays to identify traces of *A. tumida* and *G. mellonella* in honey through its eDNA. The use of honey eDNA to detect and monitor these two insects could be important to establish early and effective measures to contain these two damaging pests at the local (e.g., apiary) or regional scales.

## 2. Materials and Methods

### 2.1. Samples

A total of 79 honey samples (55 monofloral, 13 polyfloral and 11 honeydew honey samples) were provided by beekeepers or purchased from trade markets. All honey samples were produced in Italy (in 16 out of 20 regions) in the years from 2007 to 2019. One honey sample produced in Calabria was obtained from an apiary that was known to be infested by *A. tumida*. Table S1 reports the list of samples, including their origin and the year of production. Three other honey samples, obtained from honeycombs of three different colonies were from Bovo et al. [11]. These three honey samples were analysed by whole DNA shot-gun sequencing and bioinformatic analyses assigned several reads to *A. tumida* genome, even if the matches were considered spurious results derived by the presence of DNA traces from coleopters in the samples [11].

Four adult SHBs were collected from the infested apiary in Calabria region from which we also collected the honey sample mentioned above. The identification of the specimens was obtained according to the morphological method of the European Union Reference Laboratory (EURL) for Bee Health [64]. Four adult greater wax moths were collected from infested honeycombs stored by a beekeeper in Emilia Romagna region (Italy). Classification of these specimens was obtained following the dichotomous keys of Ellis et al. [57]. Four honey bees were collected from an apiary in Emilia Romagna region. All insects were brought to the laboratory in absolute ethanol and then stored at −20 °C till DNA extraction. All activities followed the Italian Regulation of Veterinary Inspection (Regolamento di Polizia Veterinaria) [65].

### 2.2. DNA Extraction

DNA was extracted from the honey samples following the protocol previously reported [12,23,24]. Briefly, 50 g of honey for each sample was divided into four 50 mL tubes (12.5 g for each tube), which were then filled with ultrapure water, vortexed and then incubated at 40 °C for 30 min. The tubes were then centrifuged for 25 min at 5000× *g* at room temperature and the resulting supernatant was discarded. To each tube, five mL of ultrapure water was added to the obtained pellet which was resuspended. The content of the four tubes was merged in one and then diluted again with ultrapure water. Another centrifugation step (25 min at 5000× *g* at room temperature) obtained a final pellet which was resuspended in 0.5 mL of ultrapure water. Then, 1 mL of CTAB extraction buffer (2% (*w*/*v*) cetyltrimethylammonium bromide; 1.4 M NaCl; 100 mM Tris-HCl; 20 mM EDTA; pH 8) and 5 μL of RNase A solution (10 mg/mL) were added to the resuspended pellet and incubated for 10 min at 60 °C. After, 30 μL of proteinase K (20 mg/mL) was added for an incubation at 65 °C for 90 min that included a gentle mixing. Samples were then cooled at room temperature and subsequently centrifuged for 10 min at 16,000× *g*. After this step, 700 μL of the supernatant was transferred into another tube containing 500 μL of chloroform/isoamyl alcohol (24:1). Then, the content was mixed by vortexing before a centrifugation for 15 min at 16,000× *g* at room temperature. The supernatant was transferred into a new 1.5 mL tube where the DNA was precipitated with 500 μL of isopropanol and then with 500 μL of ethanol 70%. The DNA pellets were rehydrated with 30 μL of sterile water and stored at −20 °C until the PCR analyses.

DNA extraction from the legs of the small hive beetles, the head of the greater wax moths and the head of the honey bees was carried out using the Wizard^®^ Genomic DNA Purification Kit (Promega, Promega Corporation, Madison, WI, USA), following the manufacturer’s instructions for animal tissues.

Extracted DNA was visually evaluated by 1% agarose gel electrophoresis in TBE 1X buffer, after staining with 1X GelRed Nucleic Acid Gel Stain (Biotium Inc., Hayward, CA, USA) and quality checked using the NanoPhotometer IMPLEN P300 (Implen GmbH, München, Germany).

### 2.3. PCR Primers and PCR Analyses

Novel PCR primers used to amplify the DNA of *A. tumida* and *G. mellonella* were designed using the Primer-BLAST tool of the National Center for Biotechnology Information (NCBI) [66] which was developed to make primers that are specific to intended PCR targets [67]. Primer Pair Specificity Checking Parameters included the use of the whole Nucleotide Collection (March 2021) setting as organisms Arthropoda or “nr” (non-redundant Nucleotide Collection), primers with a minimum of six total mismatched to unintended targets of which at least four mismatches were at the 3′-end.

The target sequence for *A. tumida* was a region of the mitochondrial genome sequence (accession number MF943248 [68]) that encompassed the cytochrome oxidase I gene (COI), that was analysed for a total of 139,780 BLAST hits. To detect the DNA of *A. tumida*, in addition to the novel PCR primer pair, we also tested the primers developed by Li et al. [51] that were used for a qualitative assay. This primer pair (Atum-3) targets a region of the COI gene. Target sequences for *G. mellonella* were two cytochrome oxidase I gene (COI) regions of the mitochondrial genome of this species (accession number KT750964 [69]), that were analysed for a total of 138651 BLAST hits. Primer sequences, amplicon size and PCR conditions are reported in Table 1. Owing to the fact that extracted DNA from honey is usually highly degraded [12,16,22,23,24], to assess the possibility of successfully amplifying the DNA fragments from honey samples, we first verified if amplification from this matrix could be obtained for *Apis mellifera* DNA [22,23,24]. Primers to amplify honey bee DNA that targeted a region of the *A. mellifera* mitochondrial genome (Table 1) were already reported [16,17].

**Table 1:** PCR primers, PCR conditions and amplified mitochondrial DNA regions.

Target Species	Primer Pair Name	Primer Sequences (5′-3′): Forward (For) and Reverse (Rev)	Size/Ta ^1^	Amplified Region
*A. tumida*	Atumida_cox1_190	For: AGCCCAGTAACTCTATGAGCA Rev: GGAATCATTGAACAAATCCGGC	190/53	COI
*A. tumida*	Atum-3 ^2^	For: CCCATTTCCATTATGTWYTATCTATAGG Rev: CTATTTAAAGTYAATCCTGTAATTAATGG	97/53	COI
*G. mellonella*	GallMelCox1_182	For: TGAACTTGGTAATCCTGGTTCT Rev: TATTATTAAGTCGGGGGAAAGC	182/58	COI
*G. mellonella*	GallMelCox1_169	For: TTTTTAGGACTTGCAGGTATGC Rev: GGGGAAATAATACTGTTCGTTG	169/58	COI
*A. mellifera*	ACM *^3^*	For: GGCAGAATAAGTGCATTG Rev: TTAATATGAATTAAGTGGGG	C 85, M 139, A 153/51	COI-COII

^1^ Size: length of the amplified fragment in bp; Ta: annealing temperature (°C) used in the PCR analyses; ^2^ Primer pair developed by Li et al. [51]. ^3^ Primer described in Utzeri et al. [16] used to test the quality of the extracted DNA from honey samples already. Size of the amplified fragment is related to the mitotype of *A. mellifera* [16].

Amplifications were performed on a SimpliAmp Thermal Cycler (Thermo Fisher Scientific, Waltham, MA, USA). Reactions were run in a total volume of 20 μL including: KAPA HiFi HotStart Mastermix (Kapa Biosystems, Wilmington, MA, USA); 10 pmol of each primer of the selected primer pair; 10–50 ng of isolated DNA. The PCR profile was the following: initial denaturation step at 95 °C for 3 min; 35 cycles of alternate temperatures (20 s at 98 °C, 15 s at the specific annealing temperature for the different primer pairs as reported in Table 1, 30 s at 72 °C); a final extension step at 72 °C for 1 min. Amplifications for the honey DNA were carried in parallel using the DNA extracted from the two targeted insects as a positive control and as a negative control, no DNA. Amplification was tested using spiked honey DNA (100 ng) with the diluted DNA of the two pests, with decreasing DNA concentration (20, 10, 5, 1, 0.1, 0.01, 0.001 and 0.0001 ng), without changing PCR conditions. Obtained amplicons were electrophoresed on 2.5% agarose gels in TBE 1X buffer and then visualized with 1X GelRed Nucleic Acid Gel Stain (Biotium Inc., Hayward, CA, USA). To confirm the results of positive honey samples, PCR was carried out at least twice for each primer pair/honey combination.

### 2.4. Sanger Sequencing

Amplicons obtained using each primer pair from the respective control DNA and the positively amplified honey samples were sequenced using Sanger sequencing, following the procedure already reported [23,24]. Obtained electropherograms were visually inspected and analysed using BioEdit Sequence Alignment Editor v7.0.5 [70] and MEGA11 [71] software. BLASTN [72] was used to confirm the assignment of the obtained sequences to the expected species and corresponding DNA region.

## 3. Results

The DNA extracted from all of the honey samples was successfully amplified with primers designed on *A. mellifera* mtDNA, indicating that the obtained DNA was suitable for PCR amplification and did not contain Taq DNA polymerase inhibitors.

**Figure 1:**
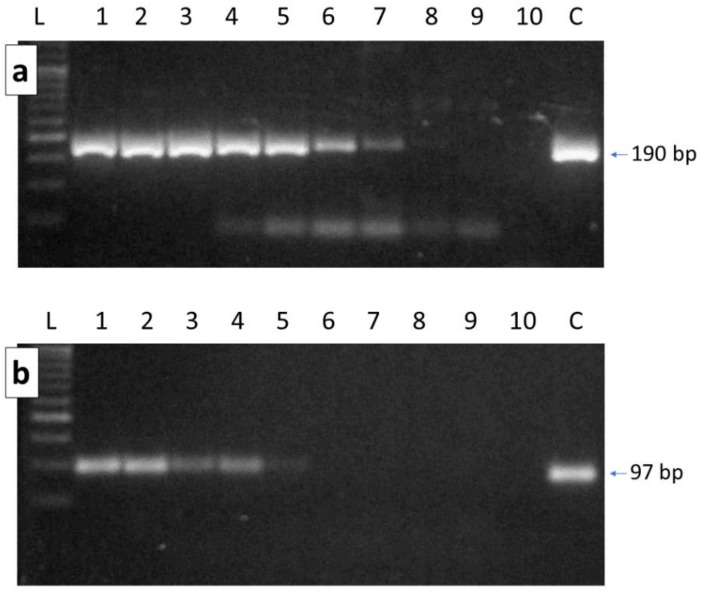
Gel electrophoresis analysis of the amplified fragments of the *A. tumida* COI regions ((**a**) 190 bp obtained from Atumida_cox1_190 primer pair; (**b**) 97 bp obtained from Atum-3 primer pair) obtained at different concentrations of the targeted template DNA. L = DNA ladder. 1: 20 ng of *A. tumida* DNA were added to the reaction mixture; which included honey DNA; 2: 10 ng of *A. tumida* DNA added; 3: 5 ng of *A. tumida* DNA added; 4: 1 ng of *A. tumida* DNA added; 5: 0.1 ng of *A. tumida* DNA added; 6: 0.01 ng of *A. tumida* DNA added; 7: 0.001 ng of *A. tumida* DNA added; 8: 0.0001 ng of *A. tumida* DNA added; 9: amplification with only the honey DNA; 10: amplification without any DNA; C: control DNA amplification (20 ng of *A. tumida* DNA) without any DNA from honey.

The novel PCR primers designed to amplify *A. tumida* mtDNA were highly specific, as expected from their in silico selection that matched only a COI region of the SHB mitochondrial genome. No amplification was obtained from the honey bee DNA or from the DNA extracted from honey samples that were previously characterized by shot-gun sequencing and that bioinformatic analyses assigned many reads to *A. tumida* genome [11]. As mentioned in that study [11], this identification from these samples was a false positive result derived by the presence in the honey of the DNA of other coleopters whose sequenced reads identified as closest matches regions of the SHB genome, even if they were not from *A. tumida*. The same results were obtained using the second primer pair tested and designed by Li et al. [51] on the COI gene region of *A. tumida*. Amplifications of the diluted crude DNA from a single SHB specimen mixed with honey DNA was used to test the sensitivity of these two primer pairs. The primer pair that we designed (Atu-mida_cox1_190) yielded positive amplifications for all dilutions (Figure 1a), including when the SHB DNA was added to the reaction mixture at the concentration of 0.0001 ng/µL, that could be considered the limit of detection (LOD). The lowest dilution, however, produced a faint band of the expected amplicon. The second primer pair that we tested (Atum-3; designed by Li et al. [51]) yielded positive amplifications at the 0.1 ng/µL dilution of the SHB DNA (Figure 1b), which could be considered its LOD. With the other dilutions (from 0.01 to 0.0001 ng/µL), this primer pair could not amplify the targeted mtDNA region, as evidenced on agarose gel electrophoresis (Figure 1b).

PCR of the DNA extracted from all commercial honey samples reported a positive amplification for both pairs for just one honey produced in Calabria in an apiary that experienced infestation of *A. tumida*. Sequencing of the obtained amplicons confirmed the expected origin from SHB. All other honey samples produced in Italy did not have any positive amplification (Table S1).

**Figure 2:**
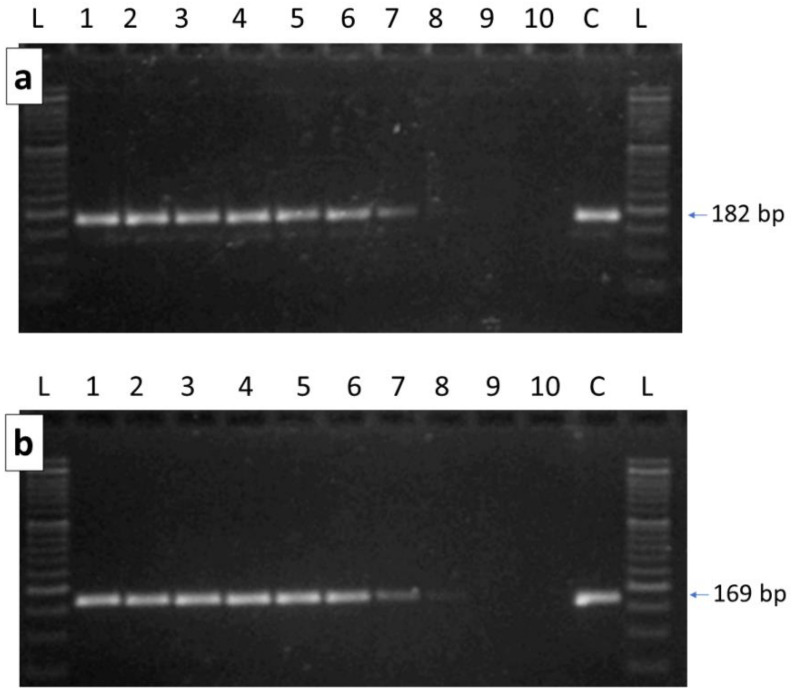
Gel electrophoresis analyses of the amplified fragments of the *G. mellonella* COI gene regions ((**a**) 182 bp obtained from GallMelCox1_182 primer pair; (**b**) 168 bp obtained from GallMelCox1_168 primer pair) obtained at different concentrations of the targeted template DNA. L = DNA ladder. 1: 20 ng of *G. mellonella* DNA were added to the reaction mixture; which included honey DNA; 2: 10 ng of *G. mellonella* DNA added; 3: 5 ng of *G. mellonella* DNA added; 4: 1 ng of *G. mellonella* DNA added; 5: 0.1 ng of *G. mellonella* DNA added; 6: 0.01 ng of *G. mellonella* DNA added; 7: 0.001 ng of *G. mellonella* DNA added; 8: 0.0001 ng of *G. mellonella* DNA added; 9: amplification with only the honey DNA; 10: amplification without any DNA; C: control DNA amplification (20 ng of *G. mellonella* DNA) without any DNA from honey.

The two primer pairs that targeted the COI gene of *G. mellonella* mitochondrial genome produced the expected fragments of 182 and 169 bp, respectively. Amplicons were obtained for all dilutions of the targeted DNA of the greater wax moth, even at the lowest concentration where, however, a faint band was obtained for both products (Figure 2). In addition, for these primer pairs, the LOD could be considered the lowest concentration of *G. mellonella* DNA that we tested (i.e., 0.0001 ng). The DNA extracted from all three honey samples derived from honeycombs gave positive amplification with both primer pairs, confirming the results derived from shot-gun sequencing that identified reads that were derived from this moth [11]. Sequencing confirmed that the obtained amplicons derived from the targeted *G. mellonella* mtDNA regions. Amplified fragments were obtained, with 100% matching results with both primer pairs, in many commercial honey samples investigated in this study: about 66% of these samples gave an amplified fragment with both specific primer pairs designed for this moth (Table S1). This result indicated that most honey samples derived from colonies where *G. mellonella* was present, suggesting that this pest is distributed on a widespread basis in Italy.

## 4. Discussion

Honey is an important source of eDNA which provides a fingerprint of the production systems and hive environments where this product comes from [10,11]. Honey eDNA can contain information on the pathogens, parasites and pests that circulate in the apiary and infect or infest the colonies [18,19,20,21,22,23,24]. In this study, we demonstrated that honey is also a useful source of DNA traces left by two important pests, *A. tumida* and *G. mellonella*, which are matters of growing concern for the apiculture sector of many regions and countries [35,36,37,48,56,60].

Honey DNA is usually highly degraded and for that reason it is possible to successfully amplify only short fragments. Therefore, we designed and tested primer pairs that targeted fragments of <200 bp, which, in our experience, can be considered a good compromise between the possibility of obtaining informative DNA sequences and assuring a successful amplification of the DNA that is possible to extract from this matrix [16,22,24]. Other primer pairs could be designed and then tested for the same purpose, targeting the two insect species that we investigated in this study or other pathogens, parasites and pests for which it would be useful to obtain information from the honey eDNA. This study further expanded the possibility to retrieve eDNA information from honey which would be useful to monitor the health status of the honey bee colonies, complementing our previous works that set up assays for other honey bee pathogens and parasites [22,23,24].

The results from the assays that we set up for these two pests can be interpreted only as qualitative (i.e., presence or absence of the amplification, and thus of the pests) even if we obtained information on the LOD. In three out of four tests, amplification was possible with a very low amount of the target pest DNA (i.e., 0.0001 ng). This indicates that the assays are quite sensitive and useful to detect incipient infestations that potentially would not be detected with other direct monitoring approaches. We are working to further improve these detection methods, also increasing their sensitivity, using qPCR and digital PCR approaches. Anyway, it would be quite challenging to relate quantitative measures obtained from the amplification reactions to the degree of infestations or presence of these two pests in the colonies/apiaries from which the honey comes from. Counting methods of the individuals of these two pests and evaluations to estimate the level/amount of traces or contaminations from them both could be eventually used to correlate the quantitative DNA measures with other parameters and then to validate quantitative measurements.

A few other studies already proposed DNA-based methods to detect *A. tumida* [49,50,51,52,53,54,55,73]. Some of them were developed to identify the presence of *A. tumida* in an apiary without direct evidence as they used hive debris, frass, swabs or even honey bees as sources of SHB DNA [49,53,54,55,73]. None of them, however, were tested to use honey as a source of *A. tumida* DNA traces. In our study, we designed PCR primers to maximize specificity to *A. tumida* considering a large database of reference sequences. Only one honey sample (produced in Calabria) out of the 79 analyzed samples contained SHB DNA, according to the positive amplification. This result, obtained from honey that derived from an apiary that had experienced *A. tumida* infestation, confirms the usefulness of the assay that we set up. It would be therefore useful to use this method to evaluate a much larger number of honey samples produced in Calabria to have a more detailed map of the areas in which this pest could be endemic, as the method has the potential to capture incipient infestations. Considering that sensitivity of this method is quite high, the use of honey DNA to monitor potential outbreaks or diffusions of the SHB could be proposed for routine surveillance in high-risk areas and to prevent the expansion of infestations. The method could be also useful to have an indirect evaluation of the actions taken to contain the infestation in areas where this pest is endemic. The cost of the assay is very low and can be implemented on a large scale and in many countries.

As far as we know, this is the first study that developed an assay to detect DNA traces left by the greater wax moth in any honey bee products. The obtained results indicated that *G. mellonella* can be considered a housekeeping pest in Italy, as was already well known. Its damaging impact, however, could potentially emerge where weak or stressed colonies are not able to control its infestation, with some possible waves of population growth derived by weather conditions [74]. It would be interesting to relate the presence of *G. mellonella* in honey produced in different seasons and eventually also monitor trends associated to climate changes. On the other hand, the identification of *G. mellonella* DNA in the honey could be an indicator of bad beekeeping practices, that did not properly sanitize and store combs [56] or that could cause the presence of many weak colonies in the apiaries, which, in turn, might not be able to control this pest.

## 5. Conclusions

In this study, using honey as source of DNA traces, we developed and tested non-invasive and sensitive assays to detect and monitor two honey bee damaging pests, *A. tumida* and *G. mellonella*. Veterinary inspection services can easily implement these methods on a large scale to increase biosecurity levels and prevent the spread of *A. tumida* outside the areas in which it is endemic or to evaluate the effectiveness of the actions applied to contain its dispersion. The presence of *G. mellonella* could be an indicator of the general health status and weakness of the colonies. It would be interesting to further exploit the application of the assays developed for the greater wax moth in this direction. This study further demonstrated the usefulness of honey as source of eDNA that contains important information that would be quite difficult to obtain in other ways and that is very relevant to protect the beekeeping sector.

## Data Availability

The data presented in this study are available in the article and Supplementary Materials.

## Supplementary Materials

The following supporting information can be downloaded at: https://www.mdpi.com/article/10.3390/vetsci9050213/s1, Table S1: List of honey samples analyzed in this study.
