# Supplementary material for: Honey Environmental DNA Can Be Used to Detect and Monitor Honey Bee Pests: Development of Methods Useful to Identify Aethina tumida and Galleria mellonella Infestations
Source: Vet Sci. 2022 Apr 27;9(5):213. doi: 10.3390/vetsci9050213 (PMC9147136; doi:10.3390/vetsci9050213)
Supplement: Supplementary file 1 [file vetsci-09-00213-s001.zip › vetsci-1559598-supplementary.pdf]

veterinary sciences

MDPI

Supplementary Material

Honey Environmental DNA Can Be Used to Detect and Monitor Honey Bee Pests: Development of Methods Useful to Identify *Aethina tumida* and *Galleria mellonella* Infestations

Anisa Ribani [1], Valeria Taurisano [1], Valerio Joe Utzeri [1] and Luca Fontanesi [1,*]

Table S1. List of honey samples analysed in this study. Samples have been listed according to the production region, from North to South of Italy.

No.	Honey sample	Region of Italy[1]	Year of production	Amplification[2]
1	Chestnut	Piedmont	2012	G
2	Chestnut	Piedmont	2018	G
3	Honeydew	Piedmont	2018	G
4	Honeydew	Valle d'Aosta	2018	G
5	Honeydew	Valle d'Aosta	2018	G
6	Honeydew	Lombardy	2018	G
7	Apple tree	Trentino-Alto Adige	2018	G
8	Rhododendron	Trentino-Alto Adige	2018	G
9	Honeydew	Trentino-Alto Adige	2018	-
10	Eucalyptus	Veneto	2018	G
11	Honeydew	Veneto	2018	G
12	Honeydew	Veneto	2018	G
13	Polyfloral	Veneto	2018	-
14	Marasca cherry tree	Friuli-Venezia Giulia	2018	G
15	Polyfloral	Friuli-Venezia Giulia	2018	-
16	Linden tree	Friuli-Venezia Giulia	2018	-
17	Linden tree	Friuli-Venezia Giulia	2018	-
18	Alfalfa	Emilia-Romagna	2018	G
19	Honeydew	Emilia-Romagna	2018	G
20	Polyfloral	Emilia-Romagna	2018	G
21	Dandelion	Emilia-Romagna	2018	-
22	Polyfloral	Emilia-Romagna	2018	G
23	Polyfloral	Emilia-Romagna	2007	G
24	Polyfloral	Emilia-Romagna	2018	-
25	Ailanthus	Emilia-Romagna	2018	-
26	Honeydew	Emilia-Romagna	2018	-
27	Polyfloral	Emilia-Romagna	2010	-
28	Polyfloral	Emilia-Romagna	2018	G
29	Acacia	Tuscany	2018	G
30	Sunflower	Tuscany	2018	G

31	Coriander	Umbria	2018	G
32	Bramble	Abruzzo	2018	G
33	Onion	Abruzzo	2018	-
34	Hawthorn	Abruzzo	2018	-
35	Sulla	Molise	2018	-
36	Honeydew	Campania	2018	G
37	Sulla	Campania	2018	-
38	Polyfloral	Basilicata	2018	G
39	Acacia	Calabria	2018	-
40	Orange	Calabria	2018	G
41	Orange	Calabria	2018	-
42	Eucalyptus	Calabria	2018	G
43	Eucalyptus	Calabria	2018	G
44	Orange	Calabria	2018	G
45	Orange	Calabria	2018	-
46	Orange	Calabria	2018	G
47	Orange	Calabria	2018	G
48	Orange	Calabria	2019	-
49	Orange	Calabria	2019	G
50	Eucalyptus	Calabria	2019	-
51	Eucalyptus	Calabria	2019	G
52	Orange	Calabria	2019	G/A
53	Orange	Calabria	2019	G
54	Orange	Calabria	2019	-
55	Dill	Sicily	2018	-
56	Orange	Sicily	2018	G
57	Orange	Sicily	2018	-
58	Thistle	Sicily	2018	-
59	Giant fennel	Sicily	2018	G
60	Polyfloral	Sicily	2018	G
61	Lemon	Sicily	2018	G
62	Citrus fruits	Sicily	2018	G
63	Polyfloral	Sicily	2018	G
64	Polyfloral	Sicily	2018	G
65	Sulla	Sicily	2018	-
66	Asphodel	Sardinia	2018	-
67	Thistle	Sardinia	2018	G
68	Thistle	Sardinia	2018	G
69	Cherry tree	Sardinia	2018	G
70	Cistus	Sardinia	2018	G
71	Giant fennel	Sardinia	2018	G
72	Lavender	Sardinia	2018	G
73	Polyfloral	Sardinia	2018	G
74	Rosemary	Sardinia	2018	G
75	Linden	Sardinia	2018	-
76	Thyme	Sardinia	2018	-
77	Honeydew	Sardinia	2018	G
78	Polyfloral	Sardinia	2018	G
79	Thyme	Sardinia	2018	G

[1]Samples that produced an amplified fragment with the primer pairs designed on the COI gene for *Aethina tumida* are indicated with "A", samples produced an amplified fragment with the primer pairs designed on the COI gene for *Gallelia mellonella* are indicated with "G", samples that did not produce any amplified fragments in all amplifications with the primer designed for these

two pest are indicated with "-". Honey sample n. 52 was the only sample collected in the Calabria area where *A.tumida* was detected.
